# Red-COLA1: a human fibroblast reporter cell line for type I collagen transcription

**DOI:** 10.1038/s41598-020-75683-5

**Published:** 2020-11-12

**Authors:** Hui Hui Wong, Sze Hwee Seet, Charles C. Bascom, Robert J. Isfort, Frederic Bard

**Affiliations:** 1 Institute of Molecular and Cell Biology, 61 Biopolis Drive, Singapore, 138673 Singapore; 2 The Procter & Gamble Company, 8700 Mason-Montgomery Road, Cincinnati, OH 45040 USA; 3 Department of Biochemistry, National University of Singapore, 21 Lower Kent Ridge Road, Singapore, 119077 Singapore

**Keywords:** Cell biology, Cell signalling

## Abstract

Type I collagen is a key protein of most connective tissue and its up-regulation is required for wound healing but is also involved in fibrosis. Control of expression of this collagen remains poorly understood apart from Transforming Growth Factor beta (TGF-β1)-mediated induction. To generate a sensitive, practical, robust, image-based high-throughput-compatible reporter system, we genetically inserted a short-lived fluorescence reporter downstream of the endogenous type I collagen (COL1A1) promoter in skin fibroblasts. Using a variety of controls, we demonstrate that the cell line faithfully reports changes in type I collagen expression with at least threefold enhanced sensitivity compared to endogenous collagen monitoring. We use this assay to test the potency of anti-fibrotic compounds and screen siRNAs for regulators of TGF-β1-induced type I collagen expression. We propose our reporter cell line, Red-COLA1, as a new efficient tool to study type I collagen transcriptional regulation.

## Introduction

The extracellular matrix (ECM) is a key contributor to the biomechanical and biochemical properties of tissues. The quantity and composition of ECM impacts important physiological processes such as development, wound healing and organ maintenance^1^. Type I collagen forms fibers that are major structural component of the dermis, contributing to 70% of dermal ECM. In addition, type I collagen can act as a ligand for receptor-mediated signalling to regulate many aspects of cellular processes downstream, including migration, cell survival and growth^2^. Type I collagen has a long half-life in tissues, estimated to be about 70 days. Hence, its production in tissues must be tightly regulated.

Fibroblasts are the archetypal cell type producing type I collagen and other ECM molecules. Under normal physiological conditions, collagen genes expression is kept at low steady rates. During wound healing, fibroblasts upregulate type I collagen transcription rates by several folds to initiate tissue repair. Yet, the timely and appropriate return to basal synthesis rates following wound resolution is critical, as excessive or prolonged synthesis leads to abnormal scar formation^3,4^.

Chronic overproduction and accumulation of type I collagen is also the underlying mechanism for fibrosis, a debilitating pathology that often results in organ damage and failure^5,6^. Fibrotic disorders are involved in nearly half of deaths in developed countries, as fibrosis plays important roles in cardiovascular and pulmonary diseases. This figure is likely to further increase with longer life spans of the human population^7^. Yet, there are no effective ways to treat fibrotic diseases. Two drugs, pirfenidone and nintedanib (Ofev) have been approved for the specific treatment of idiopathic pulmonary fibrosis (IPF). They were not developed to specifically target fibroblast or type I collagen fibrosis. Nintedanib was originally developed as an anti-angiogenic drug for cancer treatment, and later found to target tyrosine kinases involved in IPF^8,9^. The efficacy of Nintedanib to treat IPF is limited, with a partial improvement of pulmonary function and no significant increase in quality of life or survival^10^ . The mechanism of action of Pirfenidone is still largely unknown^11^. While it has been shown to have anti-fibrotic activity, it is also not a cure for IPF^12^. Other fibrotic diseases are also poorly managed. Therefore, the discovery of new targets and therapies to halt or reverse processes pertaining to fibrogenesis remains an important unmet clinical need.

On the other end of the expression spectrum, a decrease in type I collagen abundance in the dermis is the underlying cause of wrinkle formation. The decrease is driven by a reduction of type I collagen gene expression in skin fibroblasts. It remains unclear why skin fibroblasts reduce their synthetic activity, albeit aging-associated cellular senescence or photodamage by UV exposure have been suggested as underlying factors^13^. Hence, it is likely that agents promoting type I collagen synthesis will be key to future anti-aging and aesthetics treatments.

Transcriptional activation of the type I collagen genes is relatively poorly understood. It is modulated by soluble molecules, of which TGF-β1 is the most potent known. TGF-β1 is the predominant stimulatory factor of ECM synthesis in wound healing, scarring and fibrosis^14,15^. The binding of TGF-β1 to its receptor TGF-β1 type II receptor (TβRII) initiates the phosphorylation of type I activin receptor-like kinase receptor (ALKs). ALKs in turn recruit and activate the Smad proteins complex, which translocates to the nucleus^16^. The complex together with other transcriptional co-factors activates the transcription of type I collagen^17^. TGF-β1-mediated type I collagen activation can be antagonised by cytokines such as Tumour Necrosis Factor alpha, TNFα. TNFα activates nuclear factor kappa-B (NF-ĸB) signalling to suppress TGF-β1/SMAD signalling via upregulation of an inhibitory Smad protein^18^. Outside of TGF-β1 signalling cascade, few factors are known to control type I collagen levels; however, it is likely that many other soluble or insoluble, cellular or mechanical signals are involved.

While type 1 collagen is encoded by two genes, COL1A1 and COL1A2, their transcription rates are tightly coordinated^19^. Therefore, we chose to focus on the regulation of COL1A1. Current methods of assaying for type I collagen transcriptional changes primarily rely on mRNA quantification or promoter-driven reporter biochemical assays^20^. mRNA quantification requires cell destruction and is not easily multiplexed; while promoter-driven reporter assay do not capture the full transcriptional control of the COL1A1 promoter since only a fragment of the promoter region is used. In addition, if the reporter construct is integrated in the genome ex-chromosomal and aberrant chromosomal localization issues come into play. Furthermore, multi-step manipulation and expensive reagents lead to high costs, making them poorly suited for high-throughput screens. Here we describe a reporter system that is high-throughput compatible, requires minimal sample handling, and potentially allows multiple cellular features, in addition to type I collagen expression, to be simultaneously captured. Our strategy involves genetically inserting a fluorescent reporter downstream of the endogenous COL1A1 promoter in a telomerase-mediated, immortalized dermal fibroblast cell line, denoted BJ-hTERT fibroblasts. Herein, we show that our image-based assay allows for the efficient and sensitive screening for regulators of type I collagen transcription.

## Results

### Generating a knocked-in COL1A1 reporter allele using TALENs and recombination

**Figure 1:**
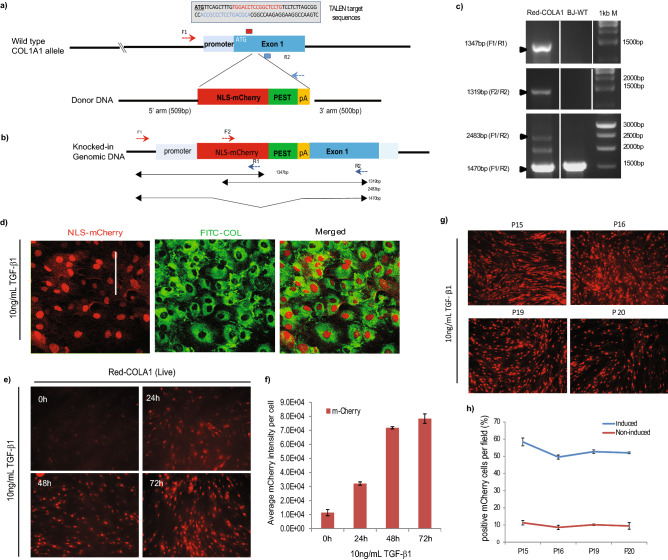
Design and generation of Red-COLA1 reporter cell line. **(a)** TALENs binding sites in COL1A1 genomic locus. TALENs recognize bases + 11 (in red) and + 43 (in blue) base pairs from the start codon (underlined) in endogenous COL1A1 genomic locus. Donor DNA cassette comprises of a nuclear localizing mCherry-reporter (NLS-mCherry) fused to a destabilising proline-glutamate-serine-threonine-rich (PEST) sequence signal, followed by polyA tail (pA) flanked by two homologous recombination arms (5′L and 3′R). **(b)** Schematic diagram of knock-in allele. Primer binding sites (F1, R1, F2, R2) and their expected band sizes for junctional PCR analysis are denoted with arrows (bottom). **(c)** Junctional PCR. Amplification of the integrated cassette was detected with 2 independent pair of primers that specifically target endogenous COL1A1 and m-Cherry sequences. Full length gels are presented in Supplementary Fig. 1e. The primer positions are indicated on the schematic of the targeted COL1A1 locus in **(a,b)**. **(d)** Expression of both mCherry and endogenous collagen (FITC stained) in Red-COLA1 reporter cells at 72 h following 10 ng/mL of TGF-β1 stimulation. Red-COLA1 cells immunostained with rabbit antibodies for type I collagen 1 (COL1) and labelled with anti-rabbit conjugated with FITC (COL1-FITC). Image acquisition (10 ×) was performed 3 days post-induction. **(e)** Representative live images of Red-COLA1 treated with TGF-β1 for 24, 48 and 72 h. **(f)** Cells were fixed at the end of the experiment and stained with Hoechst 33342 to reveal the nuclei. The intensity levels of both mCherry and nuclei count from 4 sites of 3 independent wells were calculated using the manufacturer’s analysis software and normalised to their respective untreated controls (24 or 72 h). Error bars represent SD from two independent experiments. **(g)**. Red-COLA1 derived from post FACS-enrichment were cultured for 20 passages and imaged for m-Cherry expression. Images were acquired from fixed cells at 72 h following 10 ng/mL of TGF-β1 stimulation. **(h) **Quantification of TGF-β1-responsive cells at different cell passages. Percentage of m-Cherry positive cells were calculated by applying a nuclei mask and quantifying the number of m-Cherry positive signal that overlaps with nuclei mask over the total nuclei detected.

To monitor the activity of the endogenous COL1A1 gene, we sought to insert a reporter gene after the promoter using zinc finger nucleases (*T*ranscription *a*ctivator-*l*ike *e*ffector *n*uclease, TALEN). TALENs binding sites were selected in proximity of the start codon (+ 11 and + 44 bp) (Fig. 1a). The TALENs expression constructs recognising the selected DNA sequences were generated and transfected into human U2OS cells and their cutting efficiency (66%) were verified with the standard SURVEYOR assay (See Supplementary Fig. S1a).

In order to obtain a fluorescence-based reporter cell line optimal for image-based analysis, we designed the reporter to embody several features (Fig. 1a). First, we added to the fluorescence protein, m-Cherry, an N-terminal nuclear localisation signal (NLS). This enforced subcellular localisation is meant to improve image segmentation and signal quantification. To reduce basal background levels, the m-Cherry was fused with a destabilising proline-glutamate-serine-threonine-rich (PEST) sequence signal derived from ornithine decarboxylase (ODC) at the C-terminus. This reporter gene, flanked with homology arms corresponding to approximately 500 base pairs 5′ and 3′ from the targeting locus was cloned into an expression vector to generate the donor DNA plasmid.

The pair of TALEN expression plasmids, along with the donor plasmid bearing the reporter gene were electroporated into immortalised human dermal fibroblast cell line, BJ-Tert. The electroporated cells were expanded and recombinants were enriched by FACS for m-Cherry positive cells. As the reporter gene was designed to have low expression in resting cells, cells were stimulated with 10 ng/mL TGF-β1 48 h prior to enhance the fluorescence signal for FACS sorting (Supplementary Fig. S1b). The m-Cherry positive cells (about ~ 1%) were collected and expanded (Supplementary Fig. S1c). The m-Cherry expression could be observed in the fibroblasts for up to 6 days post FACS, after which the m-Cherry signal receded back to background levels. We repeated the stimulation and FACS sorting, obtaining a population of low mCherry intensity (~ 13%) and a population of high mCherry intensity (~ 3%), which was selected and expanded for banking (See Supplementary Fig. S1d). This polyclonal pool was named Red-COLA1.

To verify proper insertion of the reporter gene, total genomic DNA was extracted from the sorted reporter cells for junctional PCR analysis. Primers targeting the m-Cherry gene and endogenous sequences flanking the targeted insertion site confirmed the insertion of the reporter immediately downstream of the promoter at exon 1 (Fig. 1b,c, Supplementary Fig S1e). While the cell line is polyclonal, detection of bands corresponding to wild type and modified allele suggests mostly mono-allelic insertion (Fig. 1c). This is confirmed by co-detection of both m-Cherry and type I collagen in most cells (Fig. 1d). The cell line also retained other essential characteristics indicative of proper collagen function: Endogenous collagen can be secreted into the culture medium (Supplementary Fig S2a) and form cross-linked fibrillar matrices in the extracellular substrate (Supplementary Fig S2b).

To test the imaging-based assay, we treated cells with 10 ng/mL of TGF-β1 and imaged m-Cherry in live cells over a course of 72 h (Fig. 1e). A threefold signal increase was detected at 24 h, rising to eightfold at 72 h (Fig. 1f).

To test the genomic stability of the m-Cherry reporter, we cultured the reporter cells for 20 passages under standard cell culture conditions (Fig. 1g) and compared the reporter cell lines at different passages for m-Cherry positive cells under TGF-β1 stimulation. At passage 15, about 60% of cells are responsive, and this proportion remains relatively stable until passage 20 (Fig. 1h).

### Reporter activity accurately depict changes in COL1A1 expression

**Figure 2:**
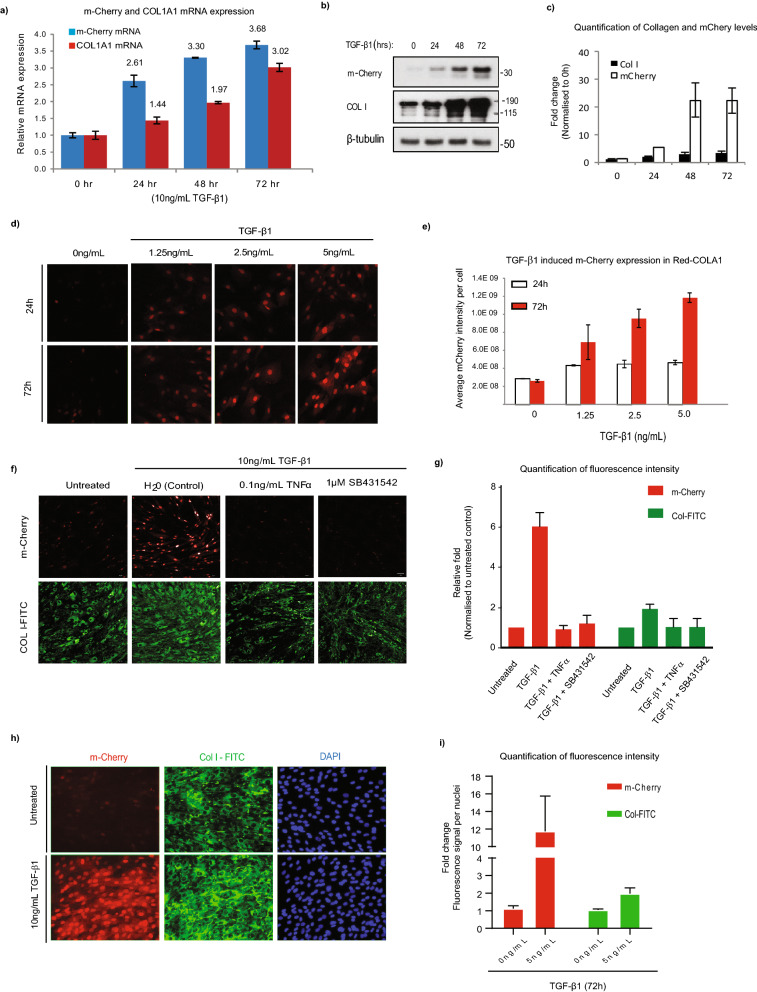
Red-COLA1 cells accurately depict changes in COL1A1 activation. **(a)** Relative mRNA expression of COL1A1 and mCherry at 24, 48, or 72 h after stimulation with10 ng/mL TGF-β1. Relative expression of the target genes was calculated with reference to the untreated control. β-actin was used as an internal control. Error bars represent SD of the average of two independent experiments performed in triplicates. **(b) **Total cell lysates (TCL) from cells treated with TGF-β1 were probed for mCherry and pro-collagen (Pro-Col I). β-tubulin was included as a loading control. Full length blots are presented in Supplementary Fig. 3**(c)**. Intensity of bands corresponding to type I collagen or m-Cherry was quantified using ImageJ and normalised with β-tubulin and expressed relative to respective 0 h control. Error bars showed standard deviation of fold-change averaged from 3 independent experiments. **(d)** Representative images of Red-COLA1 fibroblasts stimulated with 1.25, 2.5, and 5 ng/mL of TGF-β1. Image acquisition (10 ×) was performed 24 or 72 h post-induction with a high-content imager (ImageXpress) in live cells. **(e)** The intensity levels of both mCherry and nuclei count from 4 sites of 3 independent wells were calculated using the manufacturer’s analysis software and normalised to their respective untreated controls (24 or 72hours). Error bars represent SD from two independent experiments. **(f)** Representative images from untreated or TGF-β1 stimulated Red-COLA1 fibroblasts co-treated with 0.1 ng/mL of TNF or 1 µM TGF-β1 receptor inhibitor, SB431542. **(g) **Quantification of immunofluorescence in **(f)**. The intensity levels of both mCherry and type I collagen-FITC per cell, from 4 sites of 3 independent wells were calculated and normalised to untreated controls. Error bars represent SD from three independent experiments. **(h)** Untreated and TGF-β1-treated (72 h) Red-COL1A1 cells immunostained with rabbit antibodies for type I collagen 1 (COL1) and labelled with anti-rabbit conjugated with FITC (COL1-FITC). **(i)** The total intensity levels of both mCherry and type I collagen-FITC per nuclei (DAPI), 5 independent wells were calculated using the manufacturer’s analysis software and normalised to untreated controls. Error bars represent SD.

Next, we tested if m-Cherry signal in Red-COLA1 faithfully depicts changes in endogenous type I collagen expression. The reporter cells were treated with TGF-β1 over 72 h and the expression of both the m-Cherry reporter and endogenous COL1A1 alleles were compared. Both m-Cherry and COL1A1 mRNA levels were up-regulated with similar dynamics, reaching around threefold induction at 72 h post-TGF-β1 treatment (Fig. 2a). By western blot, both m-Cherry and pro-type I collagen levels increased over 72 h but contrary to type I collagen, basal m-Cherry levels were nearly undetectable (Fig. 2b,c, Supplementary Fig. 3). We verified the dynamic range of the imaging assay with a dose response to TGF-β1 at two time points (24 h and 72 h) (Fig. 2d). m-Cherry expression in Red-COLA1 displayed both dose- and time-dependent kinetics, with higher expression at 72 h (Fig. 2e).

**Figure 3:**
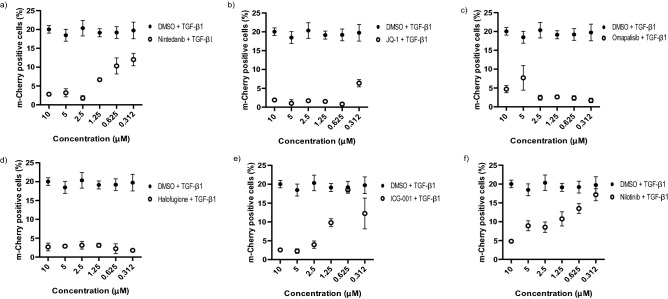
Anti-fibrotics targeting TGF-β1 signalling decrease m-Cherry expression in reporter cells. Red-COLA1 were co-treated with 10 ng/mL of TGF-β1 and decreasing concentration (10 to 0.3 μM) of **(a)** Nintedanib, **(b)** JQ-1, **(c)** Omapalisib, **(d)** Halofugione, **(e)** ICG-001 and **(f)** Nilotinib. Average percentage of m-Cherry positive nucleus were quantified and calculated, three days post treatment using manufacturer image analysis. Error bars represent SD from 4 sites performed in 6 replicate wells at each concentration.

The expression of the reporter also correlates with endogenous type I collagen when TGF-β1 signalling is disrupted. When TGF-β1 is antagonised with TNFα or with ALK5 inhibitor, SB431542, both type I collagen and m-Cherry synthesis in the reporter cells were strongly inhibited. (Fig. 2f,g).

### Red COLA1 cells allow for enhanced sensitivity and dynamic response compared to endogenous type I collagen

In culture, type I collagen expression in fibroblasts can be detected with antibodies, even in unstimulated state, contributing to high background during assay quantification. We designed our reporter to possess high turnover rate and low basal expression. We quantified and compared the expression of m-Cherry to type I collagen after 72 h of TGF-β1 induction. By image analysis, the low basal signal of m-Cherry fluorescence nuclear signal increased by 11-fold after addition of the cytokine, while type I collagen immunofluorescence revealed only a twofold increase (Fig. 2h,i). This enhanced range is also apparent by western blot (Fig. 2c).

The increase in dynamic range is likely due to the shorter half-life associated with the degradation signal tagged to the reporter protein. We generated a parallel reporter line, Red-COLA1^high^, with the same strategy except omitting the degradation signal from the m-Cherry reporter (See Supplementary Fig. S4a). Expectedly, this results in Red-COLA1^high^ possessing a higher basal expression of m-Cherry in resting cells (See Supplementary Fig. S4b). Compared to Red-COLA1 cells, the mCherry fold-change induced by TGF-β1 is more similar to that of the endogenous type I collagen protein (Fig. 2h,i; Supplementary Fig. S4c). Overall, the Red-COLA1 reporter is not only faithful in depicting changes in type I collagen gene expression, but also endowed with about fivefold increase in sensitivity.

### Use of Red-COLA1 reporter cells to test type I collagen inhibition by anti-fibrotic molecules

As transcriptional activation of type I collagen is a hallmark of fibrotic disease, we wanted to test if we could detect an effect of drugs used in clinical or pre-clinical trials against fibrosis. We treated the reporter fibroblasts with TGF-β1 together with six anti-fibrotics^9,23–27^; Nintedanib (Ofev) which is an FDA approved anti-fibrotic drug along with five other drugs currently undergoing clinical evaluation for the treatment of fibrosis including JQ-1, Omipalisib, Halofuginone, ICG-001 and Nilotinib. All six compounds inhibited TGF-β1-induced m-Cherry expression in Red-COLA1 cells at 10 μM. We re-tested these compounds at 6 serially diluted concentrations, ranging from 0.15 to 10 μM and found evidence of different potencies (Fig. 3a–f). In our assays, JQ-1, Omapalisib and Halofugione reduced TGF-β1-induced m-Cherry expression to near background levels at the concentrations tested while Nintedanib, ICG-001 and Nilotinib has an estimated IC50 of 0.625 nM, 1.25 nM and 1.25 nM respectively. Overall, the data demonstrates the effectiveness of reporter cells to detect drugs inhibiting type I collagen transcription.

### Use of Red-COLA1 cells to validate small molecules enhancing collagen expression

**Figure 4:**
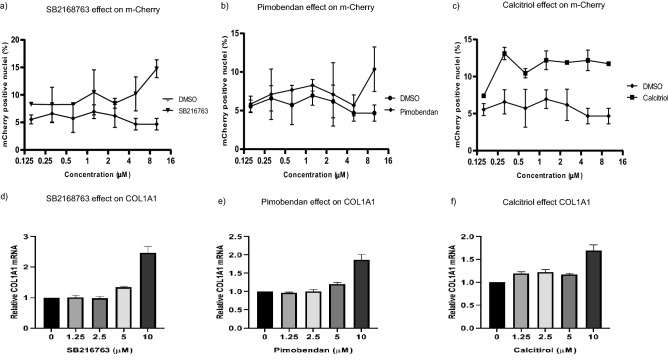
Small molecules modulating type I collagen expression. **(a)** SB216763, **(b)** Pimobendan, **(c)** Calcitirol were serially diluted and added to Red-COLA1 cells at concentrations ranging from 0.15 to 10 μM for for 3 days under standard cell culture conditions. The percentage of m-Cherry positive nucleus was calculated as previously described, and compared to control cells treated with DMSO in corresponding dilutions. Error bars represents standard deviation. **(d–f)** Relative mRNA expression of COL1A1 in cells treated with the respective compounds at the indicated concentrations. Relative expression of the target genes was calculated with reference to the DMSO-treated controls. β-tubulin was used as an internal control. Error bars represent SD of the average of three independent experiments performed in triplicates.

Glycogen synthase kinase 3β inhibitor, SB216763 and phosphodiesterase 3 inhibitor, Pimobendan have been previously reported to activate collagen I synthesis^28,29^, while Calcitriol, a vitamin D analogue, has been reported alternatively to induce or repress type I collagen expression^30–32^. We tested these small molecules in Red-COLA1 cells and found all three displayed dose-dependent induction of m-Cherry expression (Fig. 4a–c). This is consistent with the up-regulated endogenous COL1A1 transcript levels at the concentrations tested (Fig. 4d–f), confirming their stimulatory effect on type I collagen gene expression as well as the efficacy of Red-COLA1 in detection.

### Use of Red-COLA1 to identify modulators of TGF-β1 induced type I collagen synthesis

TGF-β1 stimulation of collagen expression is key event in many pathologies such as fibrosis and cancer. Understanding molecular partners of this signalling cascade could unravel novel targets for therapeutic intervention. To evaluate the use of the reporter cells in high-throughput siRNA-based screening assays, we first optimised the cells for siRNA transfection and observed that it led to non-specific red fluorescence distributed over the cytoplasm. This signal was observed with transfection reagent alone (See Supplementary Fig. S5a).

To improve signal to noise ratio in these conditions, we applied a nuclei mask and quantified nuclear m-Cherry expression rather than overall fluorescence signal (Supplementary Fig. S5b; Fig. 4b). To simplify and accelerate automated quantification, we used an intensity threshold and measured the percentage of cells with nuclear signal above threshold. Using this quantification method, we detected 4.8% of cells in the uninduced state are mCherry positive This increased to 18% following TGF-β1 stimulation, equivalent to a 4.3-fold induction over the uninduced controls (Supplementary Fig S5c-d). This approach provided improved quantification when compared to staining for endogenous type I collagen protein in the same cells, where only a 1.5-fold induction was observed (Supplementary Fig S5e).

**Figure 5:**
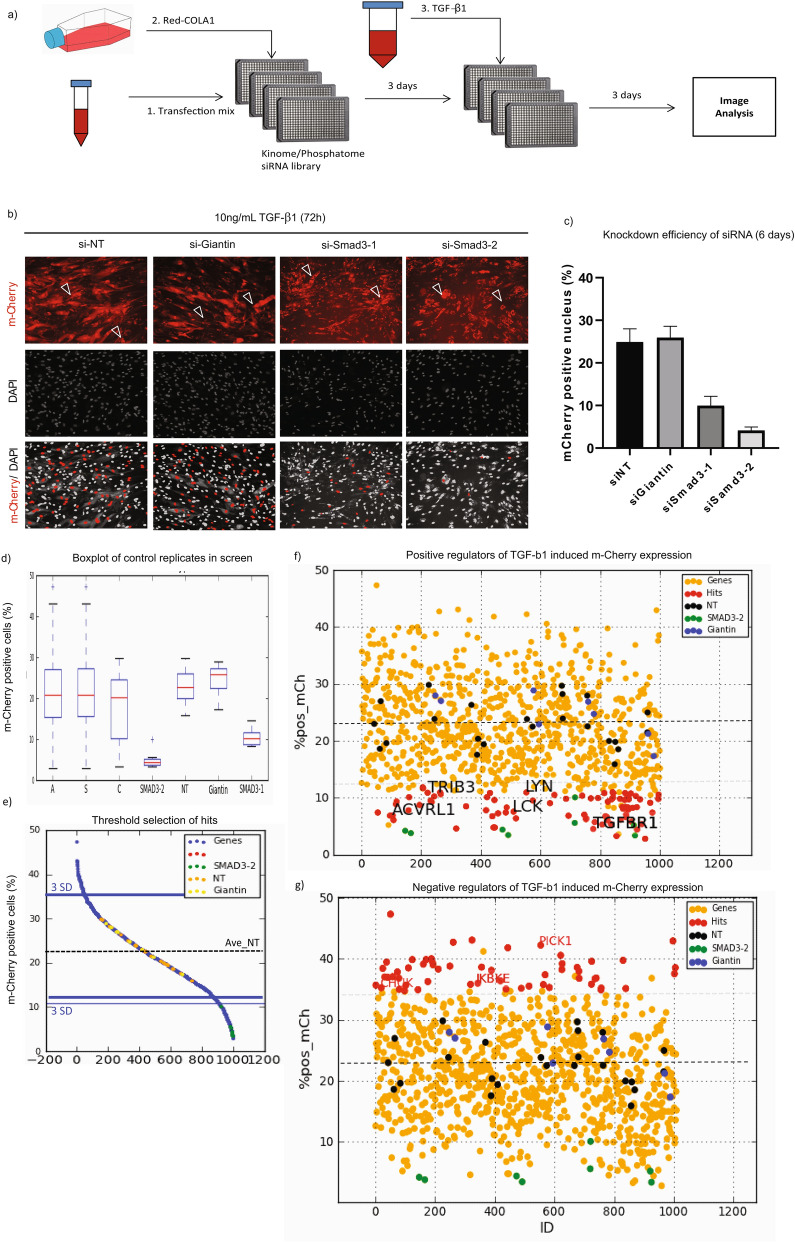
RNAi screen with Red-COLA1 identifies genes regulating TGF-β1 signalling. **(a)** Workflow of RNAI screen. Transfection reagent (RNAiMAX) was diluted in serum free media prior to dispensing into 384-well plates pre-printed with siRNA targeting genes in the human kinome and phosphatome library. Red-COLA1 cells were trypsinized, resuspended in complete growth media (DMEM, 10% FBS) and then added to wells twenty minutes after siRNA were complexed with the transfection reagent. TGF-β1 were added to wells 3 days post transfection, at a final concentration of 1 ng/mL. Image acquisition was performed 6 days post transfection. **(b)** Control siRNAs used in the screen. 2 negative control siRNAs, non-targeting siRNA (siNT), Giantin (siGaintin) and 2 positive control siRNAs targeting Smad3 were included in control wells of the screen. Non-specific m-Cherry signals observed after siRNA transfection were denoted by white open arrows. A DAPI mask was applied to quantify nuclear-localized m-Cherry signal (bottom panel) and the percentage of cells positive for nuclear m-Cherry expression above applied threshold was determined. **(c)** Average percentage of m-Cherry positive nucleus were quantified and calculated using manufacturer image analysis software. Error bars represent SD from 4 sites performed in 4 replicate wells. **(d)** Box plot of controls (si-Smad3-1, si-Smad3-2, siNT, siGiantin) and samples in the screen. A: All wells. S: Sample wells. C: Controls. **(e)** Hits identification. Sample wells with percentage of nuclear m-Cherry positive cells at least three standard deviations away (3SD) from the average of siNT (orange) controls were identified as hits. Distribution of other control wells, Giantin and Smad 3–2 are highlighted in yellow and green respectively. **(f)** Positive regulators of TGF-β1-induced m-Cherry expression. Depletion of genes that down-regulate TGF-β1-induced m-Cherry positive cells are highlighted in red. Some genes previously implicated in the pathway are indicated (ACVRL1, TGFBR1, TRIB3, LYN, LCK) **(g)** Negative regulators of TGF-β1-induced m-Cherry expression. Depletion of genes that up-regulate TGF-β1-induced m-Cherry cells are highlighted in red. Some genes previously implicated in the pathway are indicated (CHUK1, IKBKE, PICK1).

Using this strategy, TGF-β1-stimulated Red-COLA1 cells were used to screen a targeted siRNA library against 948 kinases, phosphatases and membrane receptors (Fig. 5a). siRNA targeting Smad3 were included as positive controls of TGF-β1 inhibition. Depletion of Smad3 by two independent siRNAs antagonizes TGF-β1-induced mCherry expression by more than 2.5-fold, with the second siRNA, siSmad3-2, exhibiting higher potency. This was consistent across the replicates in the screen (Fig. 5c,d). In contrast, percentage of m-Cherry positive cells depleted with Giantin (GOLB1) was indistinguishable from that of non-targeting (NT) control siRNA.

We next defined a threshold for hits. The percentage of cells with a positive nucleus was measured in siNT wells and the standard deviation between these control wells calculated. An increase by more than three standard deviations was considered significant (Fig. 5e). With this threshold, 89 genes suppressed TGF-β1-induced m-Cherry expression when depleted (Fig. 5f). Among them, TGF-β1 receptor type I (TGFBR1/ALK5) downregulated mCherry by fivefold, and was among the strongest hits. Suppression of another receptor for the TGF-β1, Activin A receptor type 1 (ACVRL1 or ALK1) led to a threefold inhibition. Our screen also picked up Tribbles homolog 3, TRIB3 (2.1-fold downregulation) as a positive mediator in TGF-β1 signalling. Our finding resonates with an earlier study identifying TRIB3 as a novel profibrotic mediator in systemic sclerosis^33^. Non-receptor tyrosine kinases, Lyn and Lck are targets of Nintedanib^9^. In the screen, depletion of Lyn and Lck reduced TGF-β1-induced m-Cherry by two- and threefold respectively.

Conversely, depletion of 55 genes augments TGF-β1-induced mCherry expression by at least three standard deviations from controls (Fig. 5g). This corresponds to at least a 50% upregulation, thus identifying genes potentially functioning as negative regulators of TGF-β1-driven collagen synthesis. One such gene is PICK1, which encodes for an adaptor protein that antagonizes TGF-β1 signalling by promoting the degradation of TGF-β1 type I receptor^34^. Loss of two NF-ĸB inhibitory proteins, IKBKA (CHUK1) and IKBKE also augments TGF-β1-induced mCherry expression by 1.5-fold, consistent with NF- ĸB signaling being opposing to that of to TGF-β1/Smad.

Taken together, the reporter cells were efficient when applied in a high-throughput setting. A total of 194 genes comprises of potentially both positive and negative regulators of TGF-β1-mediated collagen synthesis were identified (Supplementary Table 1).

## Discussion

This study reports the successful generation of a fluorescence-based reporter cell line for monitoring type I collagen transcription in dermal fibroblasts. Using TGF-β1 as a canonical regulator as well as gene depletion and small molecule treatment, we validated the ability of the system to detect modulations in type I collagen expression. Compared to the common approach of reporter systems coupled to an exogenously cloned promoter, knocking-in the reporter directly in the endogenous COL1A1 locus ensures faithfully reporting endogenous promoter activity. This is especially important as enhancers of COL1A1 genes are known to exist at distal sites from the immediate promoter^35^. One of the limitations of the cell line is that it is primarily a readout for transcriptional control of type I collagen. Materials and genes influencing post-transcriptional and post-translational modulation of type I collagen would not be captured with this assay. However, the importance of transcriptional modulation of type I collagen in many physiological and disease settings have been firmly established and it is very likely that sustained up-regulation of type I collagen levels requires transcriptional control.

One of the key features of our reporter system is the high range of responsiveness compared to assays measuring endogenous type I collagen. For instance, TGF-β1 induce a maximal increase in type I collagen protein levels of ~ twofold. This makes identifying lower potency modulators challenging. The enhanced dynamic range of detection was particularly useful in the small molecule screen for inducers of basal type I collagen expression (Fig. 5). At lower concentrations, Calcitriol, SB216763 and Pimobendan increased 10–20% of COL1A1 mRNA. These changes would not be readily picked up in high-throughput screens relying on direct COL1A1-protein based assays. Yet molecules or genes working at similar range of potency may still be valuable aesthetics and regeneration medicine.

Traditional assays quantifying type I collagen expression are often not sensitive and requires long and laborious processing, making them unsuitable for high-throughput screening. In addition, assays for type I collagen often involves destruction of cells or tissues, capturing only a static snapshot of cell activity. Our reporter assay can be used in real time imaging. One possible application includes wound healing assays in 2D or 3D settings. The assay can also be multi-plexed with other image-based assays. This is particularly useful as type I collagen expression is often co-regulated with other ECM genes in numerous physiological and disease settings.

One potential application of this cell line would be in high content screens. In recent years, several groups identified new anti-fibrotics from phenotypic cell based screens using fibroblasts features such as cell size and smooth muscle actin expression^36^. But these features may not be always correlated with type I collagen expression and fibrogenesis. For instance, while the expression of smooth muscle actin has been conventionally used as a common marker for fibrogenic cell activity, there are emerging reports that suggest they may not always correlate with severity and level of type I collagen gene expression^37^. The formation of scars owing to type I collagen overexpression, in contrast, is a unifying feature of fibrosis, despite extensive diversity in etiology. In summary, we established an in vitro model that can be effectively applied to study type I collagen transcriptional regulation. The design of cell line makes it especially useful in screens for active compounds and molecular factors modulating type I collagen expression.

## Materials and methods

Transcription activator-like effector nuclease (TALEN) pairs and donor plasmid construct. Custom TALENs were purchased from Cellectis Bioresearch (Paris, France). The left and right TALEN target sequences are 5′-TGGACCTCCGGCTCCTG-3′ and 5′- ACCGCCCTCCTGACGCA-3′, respectively. The donor plasmid was synthesized and purchased from Genscript (New Jersey, USA). The schematic of the designs of the donor plasmid is presented in Fig. 1C. In brief, the donor plasmid contained the mCherry sequence fused in frame with the mouse mTOC degradation signal (AGCCATGGCTTCCCGCCGGCGGTGGCGGCGCAGGATGATGGCACGCTGCCCATGTCTTGTGCCCAGGAGAGCGGGATGGACCGTCACCCTGCAGCCTGTGCTTCTGCTAGGATCAATGTGTAG) flanked by 500 bp of endogenous COL1A1 DNA sequences immediately upstream and downstream of the TALEN binding sequences. All plasmids were prepared using QIAGEN Maxi plasmid kit (Qiagen).

### Cell lines; cell culture media; compounds and reagents

The parental BJ-hTERT fibroblasts were provided by Procter & Gamble (Cincinnati, USA). Initially and for experiments in Figs. 1 and 2, the Red-COLA1 cell line was maintained in Dulbecco’s Modified Eagle’s Medium (DMEM) supplemented with 10% fetal bovine serum (Hyclone, Logan, UT). We later realised that to maintain their fibroblastic phenotype and better promote collagen secretion, it is advantageous to maintain the Red-COLA1 cell line in Fibroblast Basal Medium (#PCS-201-030) supplemented with Fibroblast Growth Kit-Low Serum (#PCS-201-041) with a final concentration of 5 ng/mL rh FGF basic; 7.5 mM l-glutamine; 50 µg/mL ascorbic acid; 1 µg/mL hydrocortisone hemisuccinate; 5 µg/mL rh insulin and 2% fetal bovine serum, purchased from ATCC (Manassas, VA). All experiments in Figs. 3, 4 and 5 were performed in this latter medium. All cells were grown at 37 °C in a 10% CO_2_ incubator.

Recombinant human TGF-β1 (#100-B) and tumour necrosis factor alpha (#210-TA) were purchased from R&D Systems Inc (Minneapolis, MN) and reconstituted in 4 mM HCl containing 1% bovine serum albumin and phosphate saline buffer respectively. ALK5 inhibitor, SB431542 (#4317) was purchased from Sigma and reconstituted to 10 mg/mL in DMSO as per manufacturer’s instructions. Anti-fibrotic and other compounds, Nintedanib (#S1010), Nilotinib (#1033), Omapalisib (#S2658), ICG-001 (#S2662), JQ-1 (#S7110) and Halofuginone (#S8144), Calcitriol (#1466), SB216763(#1075) and Pimobendan(#1550) were from Selleckchem (Houston, TX).

### Generation of Red-COL1A1 reporter cell line

The parental BJ-hTERT fibroblasts were electroporated using Neon Transfection System (Thermofisher) with equal amounts (3 µg) of plasmids encoding for both TALENs and the donor plasmid. Electroporated cells were expanded, and recombinant cells expressing mCherry after TGF-β1 stimulation were enriched using fluorescence activated cell sorting (FACS). The top 3% of mCherry-positive cells were gated. Sorted cells were cultured as previously described and expanded for genotyping.

### Genomic DNA extraction and genotyping

Genomic DNA was extracted with PureLink Genomic DNA mini kit (Invitrogen), and cells were genotyped using junctional PCR with the following primers: COL1A1 Endo F: 5′-ttggagaggtcctcagcatg-3′; COL1A1 Endo R: 5′-CCAGAAGTTAGCTAACCACT-3′; mCherry491 F: 5′-agatcaagcagaggctgaag-3′; and mCherry R: 5′-TCACTTGTACAGC TCGTCCATGC-3’.

### Immunofluorescence

Cells were fixed using 4% paraformaldehyde containing 2% sucrose for 15 min, washed once with D-PBS, and stained for 10 min with Hoechst 33342 diluted in D-PBS (1:10,000). The cells were then washed two more times for 5 min with D-PBS before high-throughput imaging. For selected control wells, cells were permeabilized with 0.2% Triton X-100 for 10 min and stained with type I type I collagen antibodies for 1 h, followed by anti-rabbit secondary antibodies conjugated with Alexa Fluor-488 (Invitrogen, Carlsbad, CA).

### RNA isolation and quantitative qRT-PCR

Total RNA was isolated from fibroblast cultures using an RNeasy kit (Qiagen, #74106) according to manufacturer’s protocol. RNA (1 µg) was reverse transcribed using Superscript III First-Strand Synthesis System (Invitrogen, #18080-051). Products were amplified using SYBR Green PCR Master Mix (Applied Biosystems, #4309155) on an Applied Biosystems 7500 Real-Time PCR System. The following primers were used to amplify the mCherry and COL1A1 mRNA respectively. mCherry_F: AAGCTGAAGGTGACCAAGGG mCherry_R: CAAGTAGTCGGGGATGTCGG, COL1A1_F: GTCGAGGGCCAAGACGAA, COL1A1_R: GTCTCGGTCATGGTACCT. Each reaction was performed in triplicate and data were averaged and normalized to mean β-actin RNA levels (β ACTIN_F: AGAAAATCTGGCACCACACC β ACTIN_R: AGAGGCGTACAGGGATAGCA). Fold-change in the samples was calculated using the 2-ΔΔCT method^21^.

### Western blot analysis

Whole-cell lysates were prepared by lysing cells with RIPA buffer (50 mM Tris–HCl (pH 8.0), 150 mM NaCl, 5 mM EDTA, 1% NP40, 0.5% sodium deoxycholate, 0.1% sodium dodecyl sulfate). Equal amounts of protein were loaded on 4% to 20% Tris–Glycine gels from Thermo Fisher Scientific for electrophoresis. Gels were then electrotransferred onto nitrocellulose membranes using the iBlot Dry Blotting system (Thermo Fisher Scientific) and subjected to immunoblotting using primary antibodies specific for human type I type I collagen (#1310-01) from Southern Biotech (Birmingham, AL, USA); beta-tubulin (#ab6046) and m-Cherry (#ab125096) from Abcam (Cambridge, MA). Membranes were then incubated with appropriate secondary antibodies from Abcam and subjected to enhanced chemiluminescence detection using WesternBright ECL Reagent (Advansta, USA).

### siRNAs, transfection and RNAi screening

Control siRNA, ON-TARGET plus non-targeting siRNA (#D-001810-01), and siRNA duplexes targeting Giantin (#019801) and Smad3 (#020067) were purchased from Dharmacon. The second siRNA duplex against Smad3 (#00208931) was purchased from Sigma-Aldrich (St. Louis, MO, USA). Control siRNAs were seeded in pre-determined control wells. For the screen, 2.5 µl of 500 nM siRNAs targeting the human kinome and phosphatome (Dharmacon) was robotically pre-printed onto black-walled, 384-well microplates (Greiner #781091) with Agilent Technologies Velocity 11.

Reverse siRNA transfection for each well was performed by pre-mixing 0.2 µl Therm Fisher Lipofectamine RNAiMAX with 7.3 µl Gibco Opti-MEM Reduced Serum Medium for 5 min, and dispensing the mixture into the pre-printed siRNA assay plates to form RNA-lipid complexes. All dispensing into black-walled 384-well plates (Grenier, #781091) was performed with Thermo Scientific Multidrop Combi Reagent Dispenser. Complexes were allowed to form for 20 min before dispensing 40 ul of 3000 Red-COLA1 cells into each well. At 3 days post-transfection, 10 ul of TGF-β1 was added at a final concentration of 1 ng/mL to selected control wells and incubated for another 3 days prior to fixing and immunofluorescence as described earlier. The screen was performed in duplicate.

### Automated image acquisition and analysis

Four sites (each imaged at two excitation wavelengths of 405 and 561 nm) per well were acquired sequentially at 10 × magnification on an ImageXpress Micro High-Content Imaging System from Molecular Devices. Images were analyzed using MetaXpress software (Molecular Devices). For treated cells, total cell count and total m-Cherry intensity above background were determined using the “TransFluoHT” module and average m-Cherry signal per cells of four sites were calculated for each well. For transfected cells, the percentage of cells with positive nuclear-localizing mCherry signal above background was determined using the “Translocation-Enhanced” module. The average fold-change percentage positive mCherry cells for each sample well was normalized with reference to mean of siNT (n = 8) in the same plate. Raw data were processed and analyzed using the ScreenSifter^22^ software, and the average ± SD for screen replicates were determined. The threshold for positive hits (> 2) was derived using the derivative method available in the ScreenSifter software. Similarly, the average cell count per sample well was normalized to the mean cell count in siNT wells in the same plate, with the threshold arbitrarily set to 80%.

## Supplementary information

Supplementary Information.

## Data Availability

The datasets generated during and/or analysed during the current study are available from the corresponding author on reasonable request.
