# Supplementary material for: Red-COLA1: a human fibroblast reporter cell line for type I collagen transcription
Source: Sci Rep. 2020 Nov 12;10:19723. doi: 10.1038/s41598-020-75683-5 (PMC7665053; doi:10.1038/s41598-020-75683-5)
Supplement: Supplementary file 1 — Supplementary Information. [file 41598_2020_75683_MOESM1_ESM.pdf]

Red-COLA1: A human fibroblast reporter cell line for type I collagen transcription.

Hui Hui Wong[1*], Sze Hwee Seet[1*], Charles C. Bascom[2], Robert J. Isfort[2] and Frederic Bard[1,3#]

Affiliations:

[1]Institute of Molecular and Cell Biology, 61 Biopolis Drive, Singapore 138673

[2]The Procter & Gamble Company, 8700 Mason-Montgomery Road, Cincinnati, OH 45040

[3]Department of Biochemistry, National University of Singapore, 21 Lower Kent Ridge Road, Singapore 119077

Supplementary Information

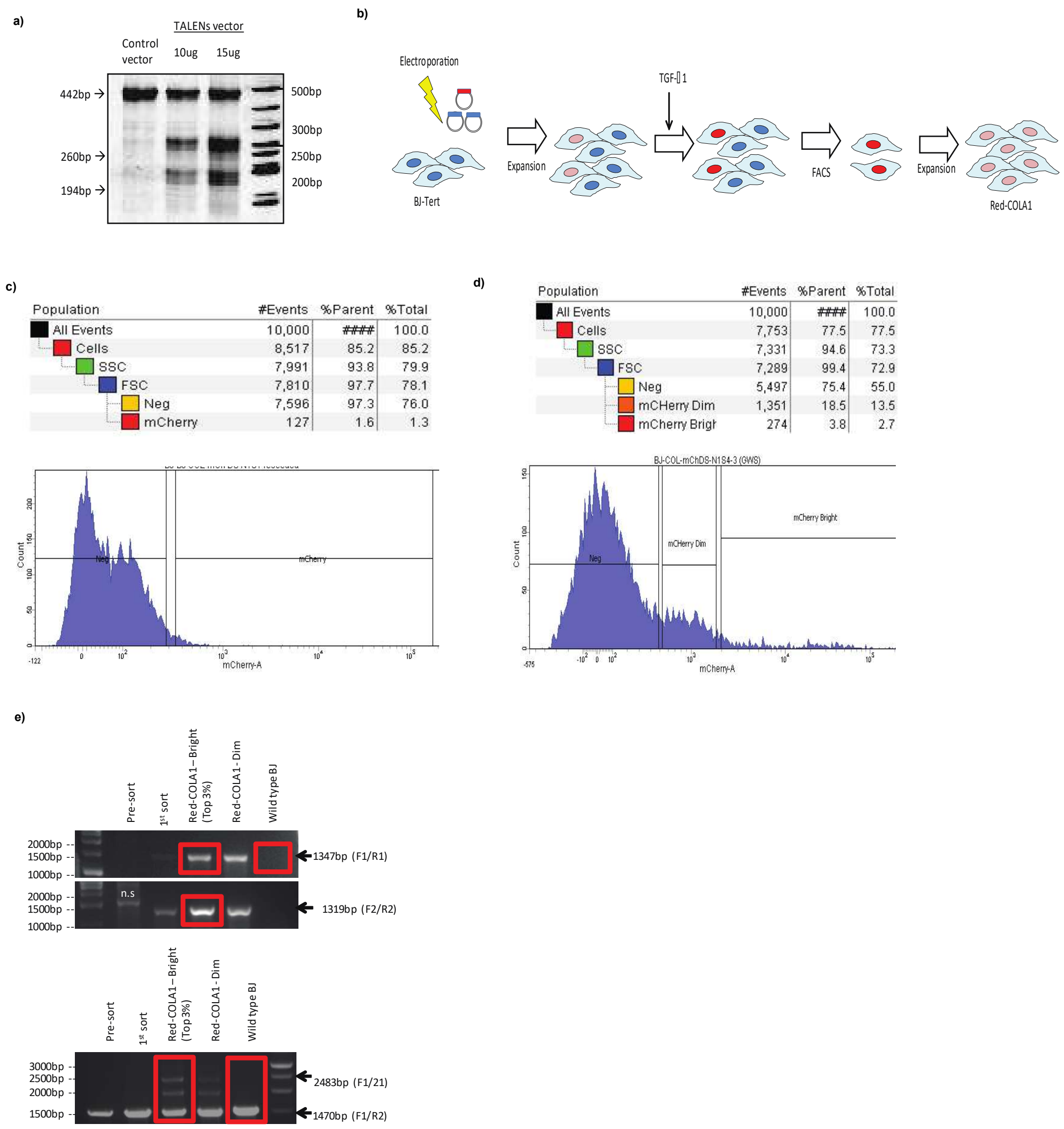

Supplementary Figure S1: Generation of Red-COLA1 reporter cell line (a) Surveyor nuclease assay. U2OS cells were transfected with control vector or 10ug and 15ug of TALENs vectors. Genomic DNA was extracted 3 days after transfection and the region flanking the binding sites (442bp) were amplified with PCR. Surveyor Mutation detection assay was then performed in accordance to manufacturer protocol. (b) Workflow of cell line generation (c) FACS gating for Red-COLA1 reporter cells after electroporation. (d) FACS gating for Red-COLA1 cells after expansion of cells. (e) Full gel used for Figure 1c. Bands corresponding to Red-COLA1 bright population and wild type BJ controls were cropped (indicated by red boxes) and presented in Figure 1c.

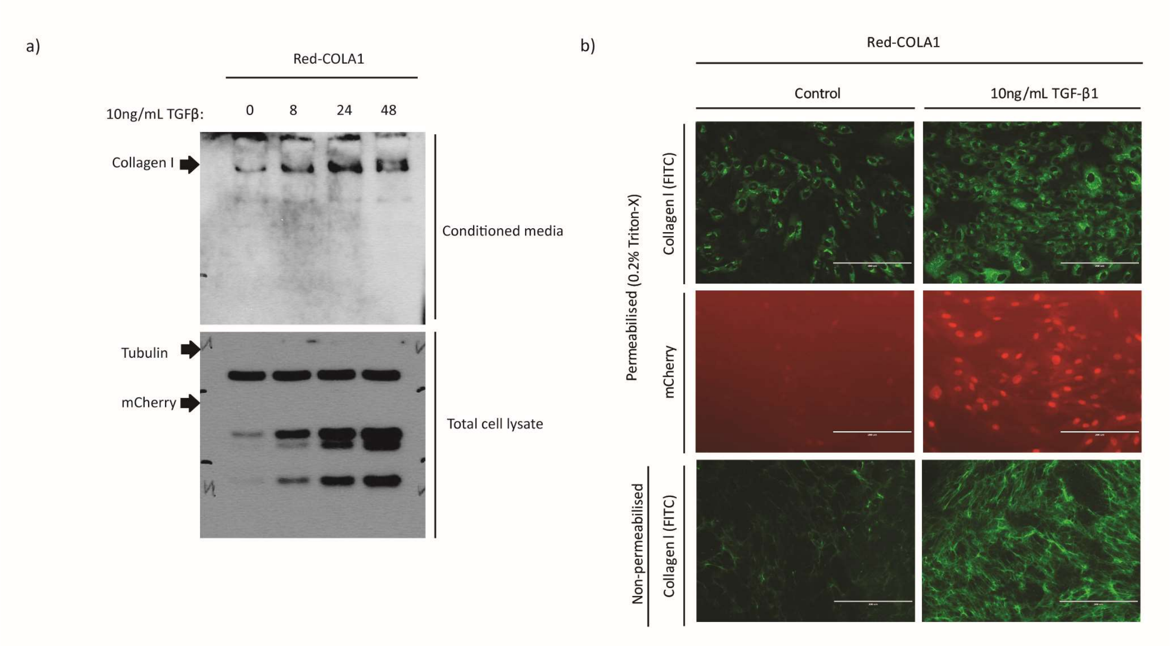

Supplementary Figure S2: Collagen I secretion and fibrillary collagen formation in Red-COLA1 cells. (a) Conditioned media of TGF-β1 treated Red-COLA1 were harvested at indicated time points and probed for collagen I. Whole cell lysate were probed for m-Cherry and β-tubulin to confirmed TGF-β1 stimulation of the reporter cells. (b) Control (untreated) and TGF-β1 treated Red-COLA1 cells were incubated for 3 days prior to immunofluorescence analysis. Cells were fixed, permeabilised and immunostained for intracellular collagen I or left un-permeabilised and immunostained for extracellular collagen I.

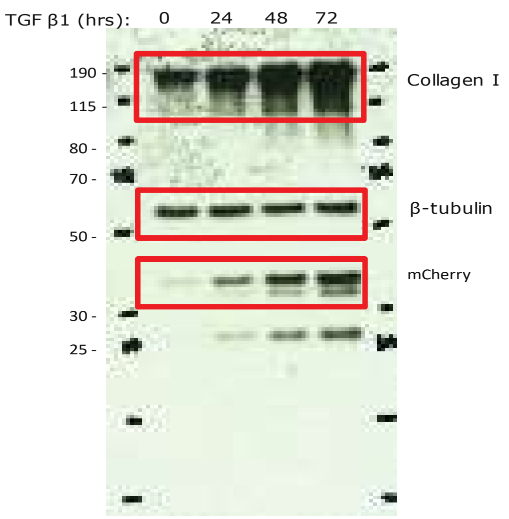

Supplementary Figure S3: Expression of m-Cherry and Collagen I expression I Red-COLA1. Shown is the full blot used to cropped out appropriate bands used for Figure 2b (indicated by red boxes).

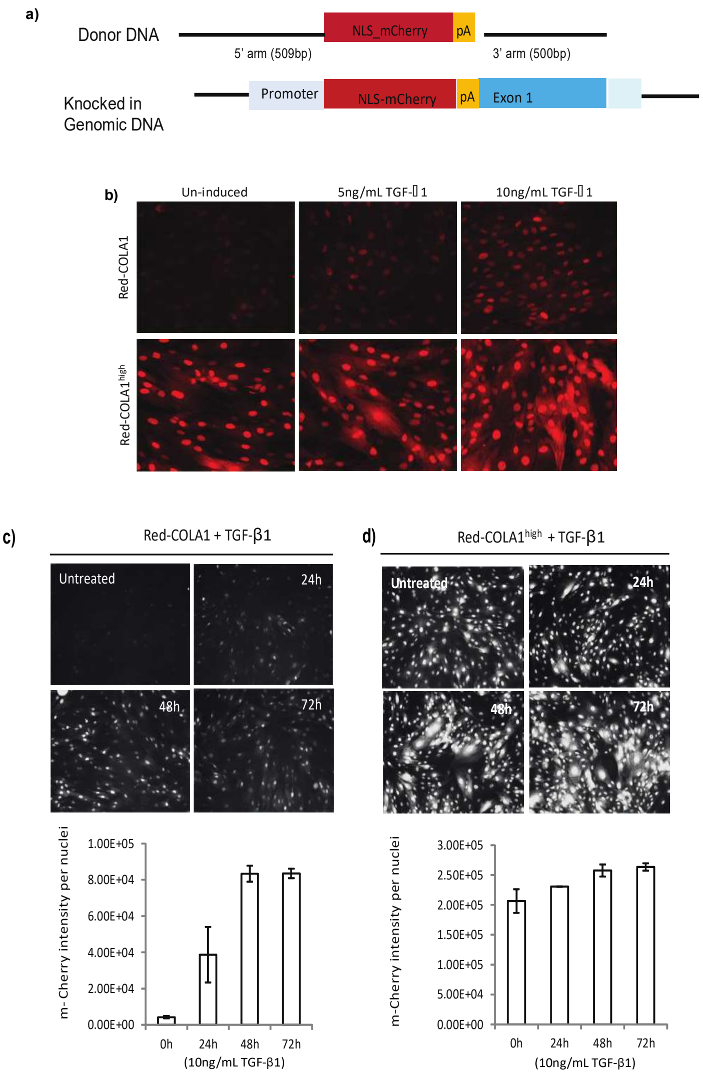

Supplementary Figure S4: Characterisation of Red-COLA1^{high} cells.

(a) Design of donor DNA plasmid for Red-COLA1^{high}. (b) Representative images of Red COLA1 and Red-COLA1^{high} (c) Quantification of m-Cherry intensity in TGF-β1-treated Red COLA1 and (d) Red-COLA1^{high}. Cells were treated with 10ng/mL of TGF-β1 for 24, 48 or 72 hours prior to fixing and image acquisition. Total m-Cherry intensity and nuclei count were quantified using the MetaExpress software and average m-Cherry fluorescence intensity per cell was calculated.

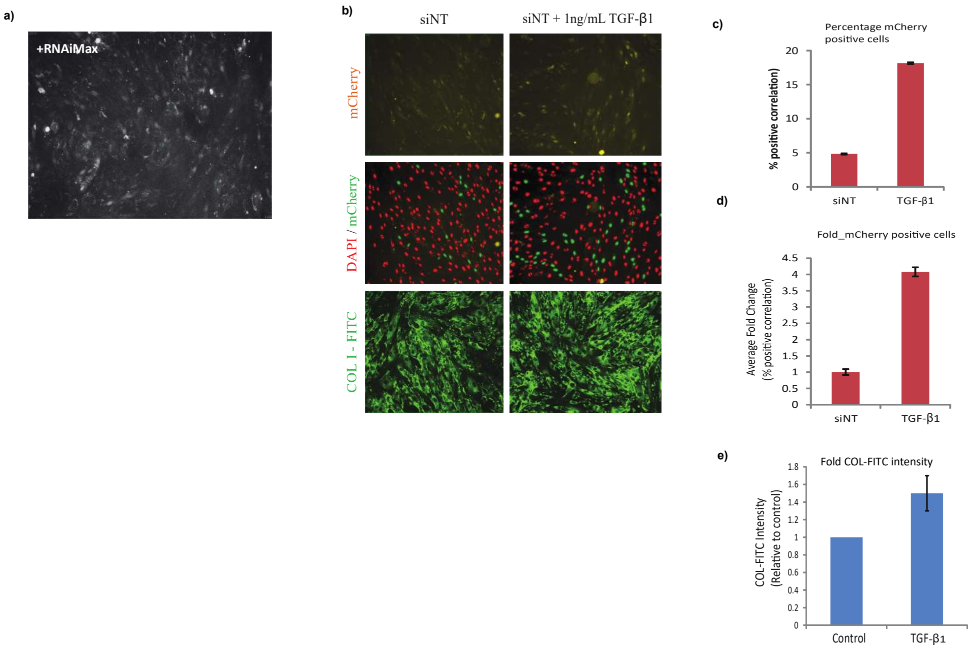

Supplementary Figure 5: siRNA transfection and image quantification in Red-COLA1.

(a) Representative image of Red-COLA1 treated with RNAiMax. (b) Red-COLA1 transfected with NT-control siRNA and treated with control (water) or 1ng/mL of TGF-β1 for 3 days were fixed, imaged for mCherry expression and immuno-stained for type I collagen expression (FITC). For mCherry, a nuclei mask revealed by Hoescht stain was used to identify cells positive for nuclear mCherry expression. (c) Percentage mCherry cells per well were scored and (d) normalised against control-treated cells using MetaExpress Software. (E) Overall intensity of Type I collagen staining revealed by FITC staining was also quantified and normalised against controls.

Supplementary Table 1: List of siRNA targeted genes downregulating mCherry expression in TGF-β1 stimulated Red-COLA1 cells

Gene_Name	Entrez_Gene_ID	Gene_Symbol	Fold_Downregulation
transforming growth factor, beta receptor 1	7046	TGFBR1	5.15
death-associated protein kinase 3	1613	DAPK3	3.86
dual specificity phosphatase 5	1847	DUSP5	3.58
protein phosphatase 1D magnesium-dependent, delta isoform	8493	PPM1D	3.32
CTD (carboxy-terminal domain, RNA polymerase II, polypeptide A) phosphatase, subunit 1	9150	CTDP1	3.23
protein tyrosine phosphatase, receptor type, O	5800	PTPRO	3.14
SCY1-like 1 (S. cerevisiae)	57410	SCYL1	3.13
alkaline phosphatase, placental-like 2	251	ALPPL2	3.08
protein tyrosine phosphatase, receptor type, B	5787	PTPRB	2.94
acid phosphatase 5, tartrate resistant	54	ACP5	2.89
toll-like receptor 4	7099	TLR4	2.83
synaptojanin 1	8867	SYNJ1	2.81
RNA guanylyltransferase and 5'-phosphatase	8732	RNGTT	2.79
protein kinase, X-linked	5613	PRKX	2.78
testis-specific kinase 2	10420	TESK2	2.75
alkaline phosphatase, intestinal	248	ALPI	2.73
apoptosis-associated tyrosine kinase	9625	AATK	2.68
protein tyrosine phosphatase, non-receptor type 2	5771	PTPN2	2.68
phosphatase and tensin homolog	5728	PTEN	2.66
WNK lysine deficient protein kinase 3	65267	WNK3	2.60
inositol 1,4,5-trisphosphate 3-kinase C	80271	ITPKC	2.59
ropporin 1-like	83853	ROPN1L	2.57
abl-interactor 1	10006	ABI1	2.52
activin A receptor type II-like 1	94	ACVRL1	2.51
sphingosine kinase 2	56848	SPHK2	2.51

Supplementary Table 2: List of siRNA targeted genes upregulating mCherry expression in TGF-β1 stimulated Red-COLA1 cells

Gene_Name	Entrez_Gene_ID	Gene_Symbol	Fold Upregulated
protein interacting with PRKCA 1	9463	PICK1	1.90907477
creatine kinase, muscle	1158	CKM	1.87
myotubularin related protein 3	8897	MTMR3	1.87
p21 protein (Cdc42/Rac)-activated kinase 4	10298	PAK4	1.84
v-raf murine sarcoma viral oncogene homolog B1	673	BRAF	1.84
mitogen-activated protein kinase kinase kinase 8	1326	MAP3K8	1.79
diacylglycerol kinase, epsilon 64kDa	8526	DGKE	1.72
FK506 binding protein 12-rapamycin associated protein 1	2475	FRAP1	1.72
PX domain containing serine/threonine kinase	54899	PXK	1.71
pyruvate dehydrogenase phosphatase isoenzyme 2	57546	PDP2	1.70
protein tyrosine phosphatase, receptor type, E	5791	PTPRE	1.69
protein tyrosine phosphatase, receptor type, J	5795	PTPRJ	1.67
discs, large homolog 3 (neuroendocrine-dlg, Drosophila)	1741	DLG3	1.66
fucokinase	197258	FUK	1.65
mitogen-activated protein kinase kinase 7	5609	MAP2K7	1.64
PCTAIRE protein kinase 2	5128	PCTK2	1.63
connector enhancer of kinase suppressor of Ras 1	10256	CNKSR1	1.63
v-erb-b2 erythroblastic leukemia viral oncogene homolog 2, neuro/glioblastoma derived oncogene homolog (avian)	2064	ERBB2	1.62
phosphoglycerate kinase 2	5232	PGK2	1.62
CaM kinase-like vesicle-associated	79012	CAMKV	1.61
calcium/calmodulin-dependent protein kinase ID	57118	CAMK1D	1.60
EPH receptor B1	2047	EPHB1	1.60
G protein-coupled receptor kinase 7	131890	GRK7	1.60
FERM and PDZ domain containing 2	143162	FRMPD2	1.60
fibroblast growth factor receptor 3	2261	FGFR3	1.58
protein phosphatase 2 (formerly 2A), catalytic subunit, beta isoform	5516	PPP2CB	1.57
chromosome 9 open reading frame 96	169436	C9orf96	1.57
phosphorylase kinase, gamma 2 (testis)	5261	PHKG2	1.57
phosphoribosyl pyrophosphate synthetase 2	5634	PRPS2	1.57

mitogen-activated protein kinase kinase kinase 10	4294	MAP3K10	1.57
phosphofructokinase, platelet	5214	PFKP	1.57
ras-related C3 botulinum toxin substrate 1 (rho family, small GTP binding protein Rac1)	5879	RAC1	1.57
p21 protein (Cdc42/Rac)-activated kinase 1	5058	PAK1	1.56
DnaJ (Hsp40) homolog, subfamily C, member 6	9829	DNAJC6	1.56
protein phosphatase 2 (formerly 2A), catalytic subunit, alpha isoform	5515	PPP2CA	1.56
protein phosphatase 2 (formerly 2A), catalytic subunit, alpha isoform	5515	PPP2CA	1.55
protein tyrosine phosphatase, non-receptor type 14	5784	PTPN14	1.55
activin A receptor, type I	90	ACVR1	1.54
protein tyrosine phosphatase, receptor type, R	5801	PTPRR	1.54
pyruvate dehydrogenase kinase, isozyme 2	5164	PDK2	1.54
mitogen-activated protein kinase 13	5603	MAPK13	1.54
casein kinase 2, beta polypeptide	1460	CSNK2B	1.53
cyclin-dependent kinase 5, regulatory subunit 1 (p35)	8851	CDK5R1	1.53
ribosomal protein S6 kinase, 90kDa, polypeptide 5	9252	RPS6KA5	1.53
dihydroxyacetone kinase 2 homolog (S. cerevisiae)	26007	DAK	1.53
guanylate kinase 1	2987	GUK1	1.53
alkaline phosphatase, liver/bone/kidney	249	ALPL	1.53
NIMA (never in mitosis gene a)-related kinase 4	6787	NEK4	1.52
calcium/calmodulin-dependent serine protein kinase (MAGUK family)	8573	CASK	1.52
protein kinase D1	5587	PRKD1	1.51
adrenergic, beta, receptor kinase 2	157	ADRBK2	1.50
cyclin-dependent kinase-like 1 (CDC2-related kinase)	8814	CDKL1	1.50
